# *MassCas* tabletop game as a training tool in mass casualty incidents for primary healthcare doctors and nurses: a pilot study

**DOI:** 10.1017/S1463423626101108

**Published:** 2026-03-26

**Authors:** Tatiana Cuartas-Alvarez, Gracia Garijo Gonzalo, Eva Valiño Otero, Cesar Luis Roza Alonso, Cecilia Naves Gomez, Rubén Villa Estébanez, Rafael Castro-Delgado

**Affiliations:** 1 Health Research Institute of the Principality of Asturias (Research Group on Prehospital Care and Disasters, GIAPREDE), Oviedo, Spain; 2 Health Service Principality of Asturias (SAMU-Asturias), Oviedo, Spain; 3 Osakidetza-Basque Health Service (Emergencias Osakidetza), Bilbao, Spain; 4 Sistema D’Emergencies Mediques de Catalunya, Barcelona, Spain; 5 University of Oviedo: Universidad de Oviedo, Spain

**Keywords:** disasters, gamification, mass casualty incidents, primary care, training

## Abstract

**Introduction:**

Mass casualty incidents (MCI) are a challenge for prehospital response. The global response may include primary health care teams (PHCT), even more in remote and rural areas. As training in MCI response is complex, it is essential to simplify it when focused in PHCT as it is a low frequency phenomenon in their context. Our objective is to measure self-perception and the impact of a brief training experience using a mass casualty incident tabletop game with primary care doctors and nurses.

**Methods:**

Descriptive study of the impact of a training intervention on 27 primary care physicians and nurses in the Principality of Asturias. A 2-h training experience was carried out using a tabletop game. Self-perception was measured using a Likert’s scale on methodology, knowledge and skills, as well as a multiple-choice knowledge test after two months. Strengths and weaknesses of the methodology were also identified using open-ended questions, as well as attitudes towards incidents with mass casualty incidents.

**Results:**

85% of participants improved their level of knowledge without providing them study material. Self-perception measured 27 items in 3 dimensions: methodology (Median = 9; interquartile range (IQR) = 2), knowledge (Median = 10; IQR = 1), and skills (Median = 9; IQR = 1). All items except one had a median greater than or equal to 9.

**Conclusions:**

Gamification using the *MassCas* tabletop game for mass casualty incidents is perceived by primary care doctors and nurses as a useful tool in their training for mass casualty incidents, as well as for acquiring specific knowledge and skills in this area.

## Introduction

A mass casualty incident (MCI) is defined by the World Health Organization (WHO) as “an event that generates more simultaneous patients than can be managed with the local resources available using routine procedures” (World Health Organization, 2007). MCIs challenge primary healthcare professionals (Bijani *et al.*, 2024) and require a coordinated response from both pre-hospital and hospital systems, generally under the supervision of an Emergency Coordination Centre (ECC).

Emergency Medical Services (EMS) traditionally develop specific procedures for MCI response. However, the role of Primary Healthcare Teams (PHCT) must also be considered (Redwood-Campbell and Abrahams, 2011), as they often collaborate with EMS and, in some cases, may arrive first, assuming leadership, organizational, and clinical responsibilities (Hodge, 1999–2000; Castro Delgado *et al.*, 2017). In many regions, PHCTs may provide the initial MCI response. For example, during the 2023 bus accident in rural Asturias involving 49 victims, the first responders were the PHCT and a Basic Life Support Unit (BLSU), initiating both organizational and clinical actions. This highlights the importance of integrating EMS and PHCT efforts in MCI situations (Castro-Delgado *et al.*, 2025).

**Figure 1.**
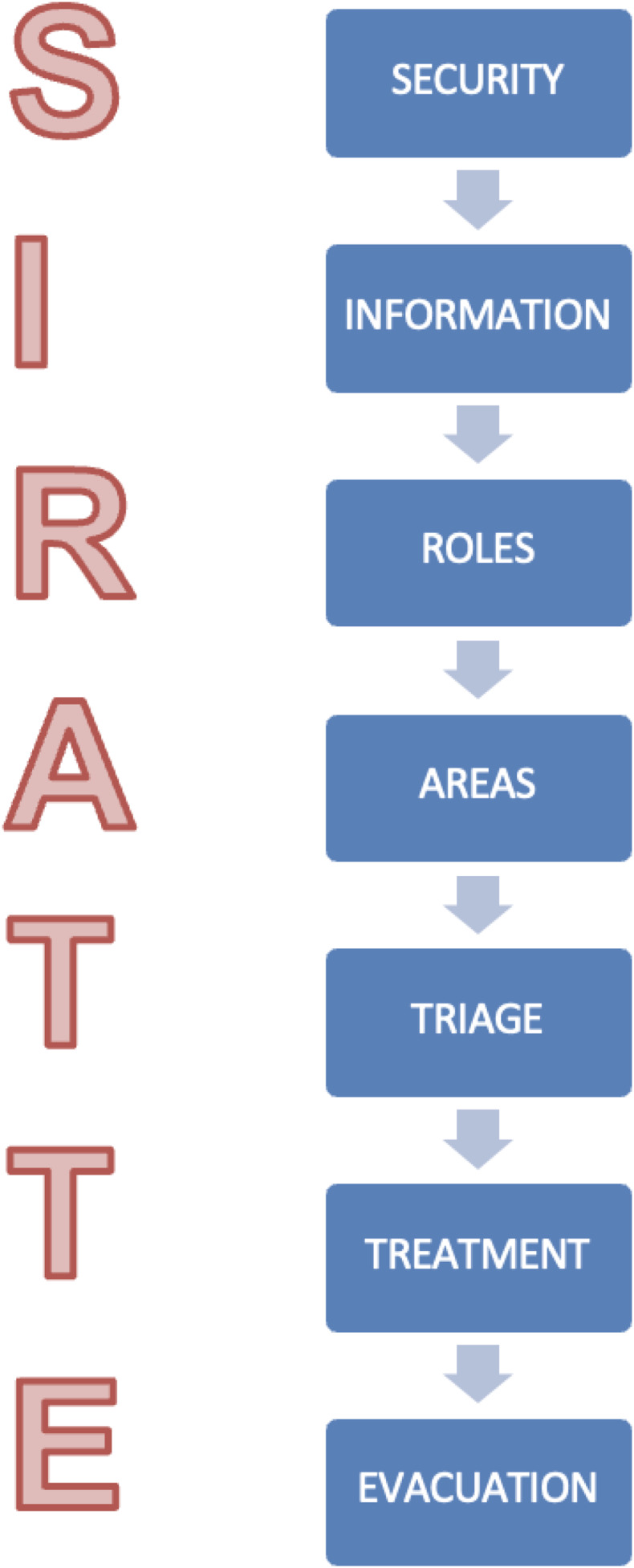
SIRATTE acronym in MCI response.

Response tasks in MCI are multiple and of different complexity depending on the training and responsibilities of the response team, but can be summarized with the acronym SIRATTE: Security, Information, Roles, Work Areas, Triage, Treatment, and Evacuation (Castro-Delgado and Cuartas-Alvarez, 2025) (Figure 1). Tools assessing the preparedness of primary care networks for MCIs and disasters have identified areas for improvement, such as interdisciplinarity, system integration, and disaster management knowledge (Lamberti-Castronuovo *et al.*, 2024). The WHO has emphasized the role of primary care – particularly in rural areas – in disaster and MCI response (World Health Organization, 2018), as it can strengthen overall health system resilience (Redwood-Campbell and Abrahams, 2011; Mawardi *et al.*, 2021).

Since PHCTs may assume organizational and clinical roles either in EMS-led responses or when arriving first, specific training is essential. MCI training has been traditionally integrated into EMS and emergency agency curricula (firefighters, civil protection, etc.), but not routinely in PHCT programs. Existing MCI training strategies (Mahdi *et al.*, 2023) often rely on complex simulations that may seem unrelated to primary care practice and can generate participant stress, affecting learning outcomes (Nieto Fernández-Pacheco *et al.*, 2018; Beerens and Tehler, 2016).

Tabletop exercises, defined by WHO as “an analog simulation that serves to manage relevant information and make key decisions… testing emergency plans in an informal and low-stress environment” (World Health Organization, 2004), have proven effective across various groups (Gauthier *et al.*, 2019; Castro Delgado *et al.*, 2022; Achatz *et al.*, 2023). However, published experiences involving primary care doctors and nurses are scarce (Fowkes *et al.*, 2010), and none have incorporated gamification techniques. Possible reasons include the historical focus of MCI training on emergency professionals and the lack of contextualization for PHCT practice.

Our training activity simulates an incident on a tabletop, allowing participants to visualize the scenario and integrate key response actions. The objective of this project is to analyse the self-perception of PC doctors and nurses regarding knowledge, skills, and teaching methodology after a short training session.

## Methodology

Quasi-experimental pretest–posttest study on PC doctors and nurses’ self-perception before and after a training intervention. Doctors and primary care nurses were recruited through the main scientific societies in this field in Asturias. There was a call to all members of the 3 medical societies and the only 1 nursing society related to Primary Health Care, and all interested doctors and nurses were recruited. This guaranteed representativeness among all different PHCT profiles.

**Figure 2.**
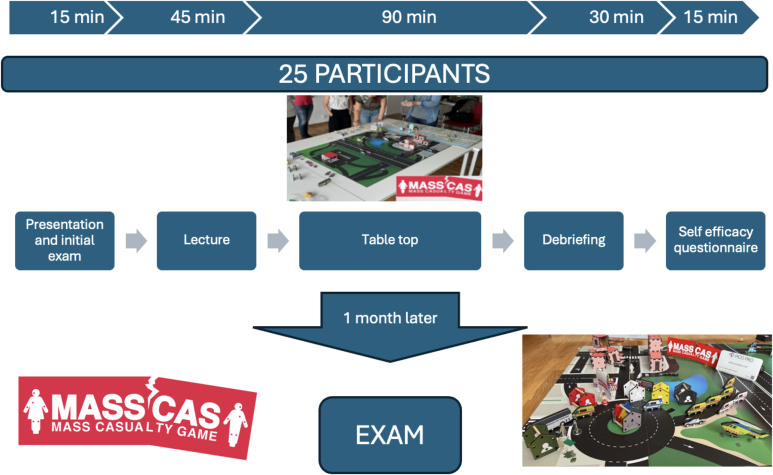
Structure of the training experience.

For this purpose, a tabletop game called “*MassCas*” was designed based on prototypes and training experiences with a tabletop tested in different groups during previous years. After the game was designed, a two-hour training action was designed, including the assessments, with the distribution shown in Figure 2. During the theoretical part, basic knowledge related to the pre-hospital response to MCI was taught following the acronym SIRATTE: Safety at the scene, Information to be transmitted to the ECC, Roles to be played, Healthcare areas, Triage, Treatment and Evacuation. The practical part consisted of developing the pre-hospital response, initially led by an PHCT, to a simulation on a table of a collapse with multiple victims affected. The key roles necessary to carry out the response to the incident were distributed among the participants. On the one hand, the simulation of a ECC that had a geographic map of the entire region where mobile response resources (Basic Life Support Unit (BLSU), Advanced Life Support Unit (ALSU), rescue teams, police, etc) and hospitals could be located, also reflected in Figure 2. The training was guided by four instructors, 2 doctors specialized in family and community medicine and two nurses. All of them doctors and nurses with several years of care experience in EMS and teaching experience in MCI.

The self-efficacy questionnaire (Annex I) consisted of 27 items distributed in 3 dimensions: perception of the methodology (10 items), perception of knowledge acquisition (12 items) and perception of skills acquisition (5 items). The items were assessed using a Likert-type scale from 1 to 10. A 10-question multiple-choice knowledge questionnaire was also administered, which was repeated after two months without providing the participants with any study material.

Three qualitative questions were also included: “Name three words that come to mind after the concept of MCI”; “Define the main strengths of the methodology”; and “Define the main weaknesses of the methodology”.

The questionnaire has previously been used in other research projects with medical students. The internal consistency of the self-efficacy questionnaire was evaluated using Cronbach’s alpha. The overall reliability of the instrument was high (*α* = 0.926). Internal consistency for the three dimensions was also satisfactory: perception of the methodology (*α* = 0.870), perception of knowledge acquisition (*α* = 0.905), and perception of skills acquisition (*α* = 0.874). These values indicate that the items within each dimension – and across the entire questionnaire – measured a coherent and reliable construct.

Paired statistical tests were used to compare pre- and post-intervention scores. As Likert-scale data are ordinal, non-parametric tests (Wilcoxon signed-rank test) were applied. Descriptive statistics were also used to summarize the data.

The open-ended responses were analysed using a conventional content analysis approach. Two researchers independently reviewed all responses and conducted an open coding process, identifying key words and recurring ideas. The codes were then grouped into broader thematic categories by consensus (organizational/coordination aspects and emotional responses). Strengths and weakness were analysed by the most repeated thematic categories. The analysis aimed to capture the most salient perceptions expressed by participants regarding the MCI concept and the educational methodology.

This study has been approved by the Drug Research Ethics Committee of the Principality of Asturias (CEImPA Code 2024.232). This study has been funded by Foundation for Research and Innovation of Principality of Asturias under the Call for Research Projects in Primary Care and Health Care 2023 (Ref: ITM-PRO-AP-CS-23-01) and led by the Research Group on Prehospital Care and Disasters.

## Results

A total of 27 primary care professionals (8 nurses and 19 doctors) participated, with an average of 11.5 years of experience in primary care (SD = 11.2) and an average of 10.1 years of experience providing urgent care in PC (SD = 10.9).

Prior to the training, they had received an average of 6.8 h (SD = 20.9) of MCI training, but if we exclude the extreme values of three participants (100, 50, and 10), we would have an average of 1 h (SD = 1.5), with 13 participants having received 0 h of prior training. Four participants had previously participated in simulation exercises on a tabletop.

When participants were asked to indicate the words that came to mind when hearing the term MCI, 123 words were obtained. The thematic analysis revealed that participants primarily associated MCI with organizational and emotional dimensions. (“*organization”, “team”, “collaboration”, “communication”, “order”, “leadership”*, etc.) and emotional aspects (“*fear”, “anxiety”, “stress”, “nervousness”, “overwhelm”, “tension”,* etc.). Among the main strengths of the methodology, participants have mentioned: “*didactic”, “entertaining”, “interactive”, “applicable”, “participatory”, “practical”, “dynamic”, “simple”* and *“visual”*. Among the main weaknesses, they have highlighted: “*excessively short duration”, “no rotation through different roles”, “little time for debriefing”* and *“excessive number of participants”*. In summary, strengths were related to the methodology’s active and practical approach, while weaknesses reflected logistical and time constraints. These results suggest that participants value experiential learning but identify opportunities for improvement in session duration and role distribution.

The results of the open questions are reflected in Table 1.

**Table 1.** Main responses to open questions

	Main answers	
What words come to mind if I talk to you about a Mass Casualty Incident?	Organizational aspects and coordination	*“organization”, “team”, “collaboration”, “communication”, “order”, “leadership”*
	Emotional aspects	*“fear”, “anxiety”, “stress”, “nervousness”, “overwhelm”, “tension”*
	Others	
What strengths have you observed in the methodology?	*“didactic”, “entertaining”, “interactive”, “applicable”, “participatory”, “practical”, “dynamic”, “simple” and “visual”*	
What weaknesses have you observed in the methodology?	*“excessively short duration”, “no rotation through different roles”, “little time for debriefing” and* “*excessive number of students”*	

In the dimension of perception of the methodology the overall median was 9 (interquartile range (IQR) = 2), in the dimension of knowledge acquisition the median was 10 (IQR = 1) and in the dimension of skills acquisition the median was 9 (IQR = 1). In the section on perception of the methodology, the increase in interest by participants in relation to the subject of study is hight (median = 10; IQR = 2), and also their perception of the usefulness of the training action for primary care doctors and nurses (median = 10; IQR = 1). In the dimension of perception of knowledge acquisition, practically all items obtain a median of 10. In the dimension of skills acquisition, all items, except one, the one related to leadership, have a median of 9. Table 2 presents the results of all items with their median and interquartile range. No significant difference was found between doctors and nurses.

**Table 2.** Results by dimensions and items

Dimension	Item	Median (1–10)	IQR
Perception Methodology (Median = 9; IQR = 2)	The methodology has increased your interest in the subject	10	2
	The methodology has facilitated the acquisition of knowledge	9	1
	The methodology has facilitated the acquisition of skills	9	2
	The methodology has increased my motivation to study	9	2
	The methodology has facilitated the acquisition of teamwork skills	9	2
	The previous explanations were adequate	9	2
	The duration of the theoretical content was adequate	9	2
	The duration of the exercise was adequate	9	3
	The methodology facilitates immersion in real cases	9	2
	I find the training experience interesting for AP professionals.	10	1
Perception acquisition knowledge (Median = 10; IQR = 1)	I have understood the different roles involved in the prehospital response to IMV	9	1
	I have understood the importance of communication between different response services.	10	1
	I have understood the organizational difficulties inherent to an IMV	10	1
	I have understood the importance of triage in an IMV	10	1
	I have understood the importance of team safety in the face of an IMV	10	0
	I have understood the importance of the chain of command to organize the response to an IMV	10	1
	I have understood the importance of communications in the response to an IMV	10	1
	I have understood the functions of the doctor in an IMV	10	1
	I have understood the functions of nurses in the face of an IMV	10	2
	I have understood the functions of the emergency medical technicians in the event of an IMV	9	2
	I have understood the functions of the rescue teams in the event of an IMV	10	2
	I have understood the functions of the security forces and bodies in the face of an IMV	10	1
Perception acquisition skills (Median = 9; IQR = 1)	The training experience has allowed me to improve my skills to coordinate multidisciplinary teams.	9	1
	My training experience has allowed me to acquire leadership skills.	8	3
	The training experience has allowed me to acquire skills in scene safety assessment	9	2
	My training experience has allowed me to acquire skills in scene sectorization.	9	1
	My training experience has allowed me to acquire triage skills.	9	1

**Figure 3.**
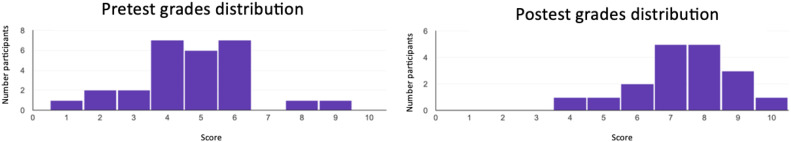
Scores distribution pre post test (2 months later).

Regarding the scores obtained in the knowledge pretest, the average score on a scale of 0–10 was 4.74 (SD = 1.74). The posttest carried out 2 months after the exercise and without study material was answered by 18 participants. The average was 7.3 (SD = 1.5). The average in the pretest of these 18 participants was 5.2 (SD = 1.4). No statistically significant differences were found between the pretest and posttest scores of doctors or nurses. Out the 18 participants who answered the posttest, 15 improved their results (Figure 3). A Wilcoxon signed-rank test indicated that post-intervention scores (Mdn = 7.5, IQR = 1) were significantly higher than pre-intervention scores (Mdn = 5, IQR = 2), *Z* = −3.26, *p* = 0.001.

## Discussion

After a short training session for MCI, PC doctors and nurses perceived it as positive in the three analysed dimensions and knowledge improves and keeps in time without providing any study material.

Primary care doctors and nurses work in multiple contexts, including emergency care (Spanish Ministry of Health, 2024). Therefore, it is necessary to improve their training and education in areas with little experience, such as MCI. In this case, actions should be chosen that have the greatest possible impact with minimum cost to be accessible to the largest number of professionals. In our study, a brief training action using a tabletop game improves PC doctor and nurses’ knowledge related to MCI. Given that the response to MCI requires knowledge and skills, we have observed that self-perception of these after the training action gets very good results. Because gamification is a teaching methodology that is not usually used in the continuing training and education programs of health professionals, we have also evaluated the perception of the methodology. Tabletop exercises can be an appropriate tool for professionals to integrate into their continuing training (Wang *et al.*, 2024), since the perception of the methodology has been very good. The teaching tool used has been recognized with the quality seal ITEMAS: The Platform for the Dynamization and Innovation of the Industrial Capabilities of the National Health System and its Effective Transfer to the Productive Sector (ITEMAS) is one of the platforms of the Carlos III Health Institute (ISCIII) to support R&D&I in Biomedicine and Health Sciences under the Spanish Ministry of Health and Ministry of Science, Innovation and Universities. This quality recognition shows that innovation in health should not only be based on complex technological aspects.

The fact that MCI represents a stressful and complex situation for PC doctors and nurses, makes it even more necessary to approach training from a playful perspective that allows them to acquire the necessary skills and knowledge. This teaching method has already been used by other authors with medical students, obtaining good results (Aster *et al.*, 2024). The general perception of the participants, in relation to the training process, identifies some strengths that are mainly related to learning in a fun, simple, participatory and very visual environment that allows them to acquire a series of knowledge and valid responses to a MCI. The fact that, despite achieving very positive results and self-perception, they consider the exercise to have been short, makes us think that the training experience has been positive. In subsequent exercises we should allow participants to play different roles, lengthening the duration and carrying out a longer debriefing that allows learning thanks to a reflective analysis of decision-making process and its repercussions on incident management.

Although the scores obtained range between 9 and 10 in all dimensions, the acquisition of skills scores slightly below the assessment of knowledge. Probably, with a longer duration of the exercise they would have had a higher self-perception in relation to the acquisition of skills. These skills that the participants perceive to have improved are of vital importance in the response to a MCI, such as team coordination, leadership or specific techniques such as triage.

Keeping the knowledge acquired two months after the training exercise represents an important strength. The results obtained indicate that the exercise got to reinforce the preparation of healthcare professionals and primary care providers to respond in the acute phase of an MCI, something that would be part of their roles in certain circumstances according to other authors (Willson *et al.*, 2021). Given the simplicity of this training experience and the logistical ease of carrying it out, we understand that it could be useful to comply with the recommendations that training should be periodic to maintain skills that are rarely used in daily work (Weinstein *et al.*, 2023). Some of the key points of our study are that mass casualty incidents are a challenge to the health response that requires the participation of different groups and structures, both healthcare and non-healthcare, and that primary healthcare teams can play an important role in the response, either as first responders or as part of a more comprehensive response with other health services. Also, training for mass casualty incidents is complex, and tabletop exercises can be a training tool, allowing the acquisition of skills and knowledge in a playful environment. *MassCas* game is a gamification training tool that allows Primary healthcare professionals to acquire knowledge and skills after a brief training intervention. We have found that primary healthcare doctors and nurses perceive an improvement in their knowledge and skills to respond to a mass casualty incident following a brief training intervention with a *MassCas* tabletop exercise. Because of this, we consider that playing with *MassCas* tabletop exercises allows primary healthcare doctors and nurses to increase their knowledge related to mass casualty incidents after a brief training session without having study material. Our training tool and strategy has a great potential in low- and middle-income countries where complex methodologies like big simulations or tabletop exercises (or even technological tools like augmented/virtual reality) are not feasible for their complexity to develop. Also, for PHCT may benefit as their learning objectives should be realistic and adapted to their needs. Key points of our research are described in Table 3.

Limitations of this study include the fact that it was conducted with a small number of doctors and nurses in a specific region with a prior interest in participating in this training experience, which could have improved the final results of self-perception. Our results may not be applicable in other contexts or led by other trainers

Because of these limitations, this study should be interpreted as a pilot experience. The sample was limited to 27 primary care professionals from a single region, which restricts the generalizability of the findings. However, the objective was not to provide definitive evidence of effectiveness, but to explore the feasibility, acceptability, and potential educational impact of a gamified tabletop intervention for MCI tailored to PHCT. These preliminary results support the relevance of this approach and justify further research. Future studies should include larger samples, involve multiple regions and healthcare systems, and assess reproducibility when delivered by different instructor teams. Multi-center validation, ideally using controlled or comparative designs and incorporating objective performance measures, will be essential to confirm the effectiveness and scalability of the *MassCas* tabletop game across diverse primary care contexts.

Gamification using the *MassCas* tabletop game for mass casualty incidents is perceived by primary healthcare doctors and nurses as a useful tool in their training in MCI, as well as for acquiring specific knowledge and skills in this area. Participants improved their average knowledge test scores after 2 months without studying any teaching material and simply by learning during the training experience. Our experience may be of use to be replicated in other contexts.

**Table 3.** Key points

What is known about the subject	Mass casualty incidents are a challenge to the health response that requires the participation of different groups and structures, both healthcare and non-healthcare.
	Primary healthcare teams can play an important role in responding to mass casualty incidents, either as first responders or as part of a more comprehensive response with other health services.
	Training for mass casualty incidents is complex, and tabletop exercises can be a training tool, allowing the acquisition of skills and knowledge in a playful environment.
What this study brings	*MassCas* game is a Gamification training tool that allows Primary healthcare professionals to acquire knowledge and skills after a brief training intervention.
	Primary healthcare doctors and nurses perceive an improvement in their knowledge and skills to respond to a mass casualty incident following a brief training intervention with a *MassCas* tabletop exercise.
	Gamification through *MassCas* table top exercises allows primary healthcare doctors and nurses to increase their knowledge of mass casualty incidents after a brief training session without having study material.

## Data Availability

Data is available upon request to the corresponding author.
